# Trilinolein, a Natural Triacylglycerol, Protects Cerebral Ischemia through Inhibition of Neuronal Apoptosis and Ameliorates Intimal Hyperplasia via Attenuation of Migration and Modulation of Matrix Metalloproteinase-2 and RAS/MEK/ERK Signaling Pathway in VSMCs

**DOI:** 10.3390/ijms232112820

**Published:** 2022-10-24

**Authors:** Yuh-Fung Chen, Kuo-Jen Wu, Lian-Ru Siao, Huei-Yann Tsai

**Affiliations:** 1 Department of Pharmacology, China Medical University, Taichung 404333, Taiwan; 2 Department of Pharmacy, China Medical University Hospital, Taichung 404332, Taiwan

**Keywords:** trilinolein, cerebrovascular diseases, cerebral ischemia/reperfusion, intimal hyperplasia, PDGF-BB, Ras/MEK/ERK signaling pathway, MMP-2

## Abstract

Cerebrovascular disease is one of the leading causes of disability and death worldwide, and seeking a potential treatment is essential. Trilinolein (TriL) is a natural triacylglycerol presented in several plants. The effects of TriL on cerebrovascular diseases such as cerebral ischemia and carotid stenosis have never been studied. Accordingly, we investigated the protection of TriL on cerebral ischemia/reperfusion (I/R) and vascular smooth muscle cell (VSMC) migration in vivo and in vitro. The cerebral infarction area, the intima to media area (I/M ratio), and proliferating cell nuclear antigen (PCNA)-staining of the carotid artery were measured. Platelet-derived growth factor (PDGF)-BB-stimulated A7r5 cell migration and potential mechanisms of TriL were investigated by wound healing, transwell, and Western blotting. TriL (50, 100, and 200 mg/kg, p.o.) reduced: the cerebral infarction area; neurological deficit; TUNEL-positive apoptosis; intimal hyperplasia; and PCNA-positive cells in rodents. TriL (5, 10, and 20 µM) significantly inhibited PDGF-BB-stimulated A7r5 cell migration and reduced matrix metalloproteinase-2 (MMP-2), Ras, MEK, and p-ERK protein levels in PDGF-BB-stimulated A7r5 cells. TriL is protective in models of I/R-induced brain injury, carotid artery ligation-induced intimal hyperplasia, and VSMC migration both in vivo and in vitro. TriL could be potentially efficacious in preventing cerebral ischemia and cerebrovascular diseases.

## 1. Introduction

Stroke, one of the cerebrovascular diseases, is the world’s second-leading cause of death and the third-leading cause of combined death and disability [1,2,3]. Among many causes of stroke, atherosclerotic stenosis or occlusion of a major cerebral artery plays a crucial role [4]. The obstruction of blood flow in a major cerebral vessel, if not resolved within a short period, will lead to a core of severely ischemic brain tissue injury [5,6].

Atherosclerosis or blood vessel injury usually results in intimal thickening, and balloon angioplasty is used in severe narrowing carotid arteries [7]. After balloon injury, vascular smooth muscle cell (VSMC) migration is essential in restenosis and impacts the final size of intimal thickening [8]; VSMCs migrate from the membrane into damaged vessels’ intima, thus inducing intimal hyperplasia [9]. Numerous growth factors and proinflammatory cytokines are involved in forming neointimal hyperplasia, and platelet-derived growth factor (PDGF)-BB plays an essential role [10,11]. PDGF-BB and its receptor are abundantly expressed in VSMCs [12,13] and significantly upregulated and activated at vascular injury sites [14,15], which activates downstream signaling pathways in VSMCs for cell proliferation and migration [16]. The breakdown of the extracellular matrix causes VSMC migration, and the possible mechanism is via the secretion of matrix metalloproteinases (MMPs) [17,18]. In addition, MMPs are increased at the site of vascular injury, and inhibitors of MMPs attenuate VSMC migration after vascular injury both in vivo and in vitro [19]. To date: vascular injury can only be controlled by medication or surgery; restenosis can occur months or years after injury and initial treatment [20]. In treating cerebrovascular diseases, a medicine that reduces VSMC migration would be beneficial. 

**Figure 1:**
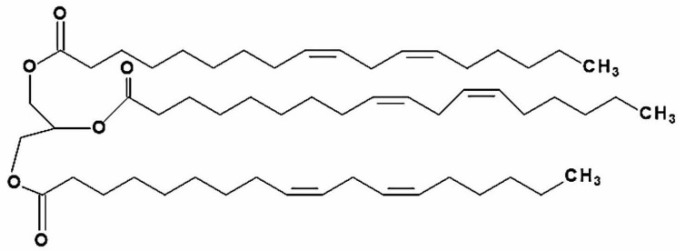
Chemical structure of trilinolein (9.12-Octadecadienoic acid (Z.Z)-1.2.3-propanetriyl ester; PubChem CID 5322095; TriL).

Trilinolein (9.12-Octadecadienoic acid (Z.Z)-1.2.3-propanetriyl ester; TriL) is a natural triacylglycerol with linoleic acid as the fatty acid residue in all three esterified positions of glycerol. The chemical structure of TriL is shown in Figure 1. TriL is presented in medicinal plants such as *Panax notoginseng*, *Panax pseudoginseng*, *Angelica sinensis* [21,22], and other origins [23,24]. TriL possesses several pharmacologic activities, such as in vitro antioxidant [25], myocardial [21], and cardiovascular protective effects [26,27]. Moreover, TriL shows anti-inflammation in LPS-treated mouse macrophages (RAW264.7) [28]. The above findings reveal that TriL is protective against cardiovascular diseases. However, the protective potential of TriL on cerebrovascular diseases such as cerebral ischemia-induced brain injury has not yet been studied. This present study aimed to evaluate the protective effects of TriL in models of ischemia/reperfusion (I/R)-induced cerebral infarction, carotid artery ligation-induced intimal hyperplasia in vivo, and PDGF-BB-stimulated VSMC migration in vitro.

## 2. Results

### 2.1. TriL Reduced Cerebral Ischemia-Induced Brain Injury in Rats

**Figure 2:**
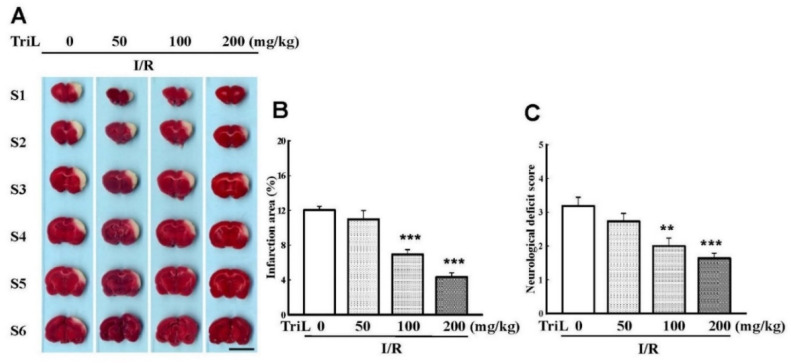
TriL reduced cerebral infarct area and neurological deficits of ischemia/reperfusion (I/R) rats. (**A**) Effects of TriL treatment (50, 100, 200 mg/kg p.o.) on cerebral infarct area at 24 h after reperfusion. The pale area represents infarct tissue, and the red-purple area normal tissue. Scale bar = 1 cm. (**B**) Infarction area by TTC staining. (**C**) Neurological deficit score. Each vertical bars represented mean ± S.E. ** *p* < 0.01, *** *p* <0.001 compared with the I/R group (n = 5).

The chemical structure of TriL is shown in Figure 1. Both common carotid arteries and the middle cerebral artery were blocked for 90 min and then reperfused for 24 h. After transcardiac perfusion, the rat brain was removed, coronally sectioned into slices, and stained with TTC. After staining with TTC, the infarction areas were visibly white and red-purple in non-infarction areas. TriL treatment (50, 100, and 200 mg/kg, p.o.) significantly reduced cerebral infarction compared with the control group (Figure 2A). Percent reduction in infraction area by TriL treatment (100, and 200 mg/kg, p.o.) were 42.45%, and 64.05%, respectively (*** *p* < 0.001) (Figure 2B). The neurological deficit score in rats were 3.18 ± 0.29, 2.72 ± 0.20, 2 ± 0.21, 1.6 ± 0.21 for control group and TriL treatment (50, 100, and 200 mg/kg, p.o.), respectively. The reduction rate was 37.14% and 48.57% by TriL (100, and 200 mg/kg, p.o.), respectively (** *p* < 0.01, *** *p* < 0.001) (Figure 2C). 

**Figure 3:**
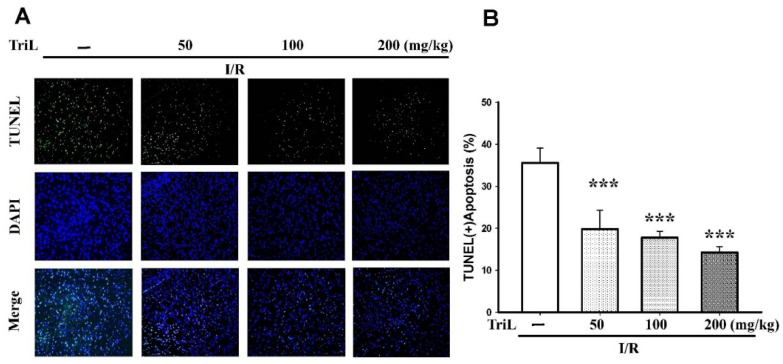
TriL extenuated ischemia/reperfusion (I/R)-induced brain damage in rats. (**A**) The presence of neuronal apoptosis within the cortex areas was determined using immunofluorescence staining of TUNEL/DAPI. Representative images of cells were stained with TUNEL (green), and the nucleus was stained with DAPI (blue). TriL treatment (50, 100, and 200 mg/kg, p.o.) significantly reduced apoptotic cells compared with the control group. (**B**) Percentage of apoptotic cells in the brain of I/R rats. TriL significantly reduced apoptotic cells compared with the control group (*** *p* < 0.001) (n = 5). Original microscope magnification = 200×.

The presence of neuronal apoptosis within the cortex areas was determined using immunofluorescence staining of terminal deoxynucleotidyl transferase mediated dUTP nick end labeling (TUNEL) assay and 4′,6-diamidino-2-phenylindole (DAPI) staining (TUNEL/DAPI). TriL treatment (50, 100, and 200 mg/kg, p.o.) significantly reduced TUNEL (+) apoptotic cells compared with the control group (Figure 3A), and the reduction rate of TUNEL (+) apoptosis was 44.37%, 50.22%, and 59.96%, respectively (*** *p* < 0.001) (Figure 3B). 

### 2.2. TriL Alleviated Carotid Ligation Induced Intimal Hyperplasia in Mice

**Figure 4:**
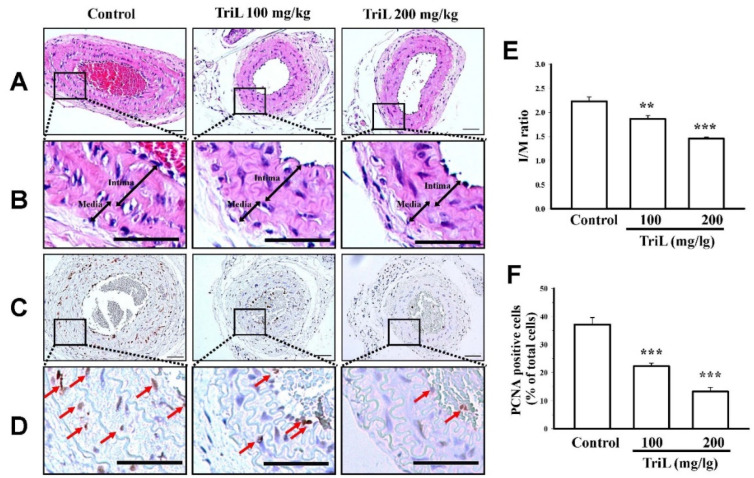
Effect of TriL on carotid artery ligation induced intimal hyperplasia in mice. After carotid-ligation, mice received oral administration of saline or different doses of TriL (100 and 200 mg/kg) once daily for 28 days. Intimal hyperplasia morphology was assessed by measuring the total lumen area, intima area (I), media area (M), and intima/media ratio (I/M). (**A**,**B**) represent photomicrographs of hematoxylin-eosin (H-E) staining, and (**C**,**D**) represent PCNA-immunoactivity staining of arterial section (200×). The arrow indicated the PCNA-positive cell. (**E**) I/M ratio. (**F**) PCNA-positive cells per total cells. Scare bar = 50 mm. ** *p* < 0.01, *** *p* < 0.001 compared with the control (saline) group (n = 5).

Mice were treated with TriL (100 and 200 mg/kg, p.o.) or distilled water (control group) for four weeks after carotid ligation. The carotid artery’s total lumen area, intimal area (I), medial area (M), and I/M ratios were determined. At 4 weeks following carotid-ligation, the I/M ratio was increased (I/M ratio = 2.22 ± 0.15). Mice treated with TriL (100 mg/kg and 200 mg/kg p.o.) showed a significant reduction in carotid intimal hyperplasia compared with the control (saline) group (Figure 4A,B). The I/M ratio was 1.86 ± 0.10 (100 mg/kg, ** *p* < 0.01) and 1.45 ± 0.05 (200 mg/kg, *** *p* < 0.001) (Figure 4E). The average inhibitory rate of the I/M ratio was 16.38% (100 mg/kg) and 34.70% (200 mg/kg) compared with the control group, respectively (Figure 4E). Arterial sections stained to detect the proliferating cell nuclear antigen (PCNA)-positive cells are shown in Figure 4C,D. The percentages of PCNA positive cells per total cell were 37.15 ± 1.75% in the control group, 22.42% ± 2.40%, and 13.27% ± 2.69% in TriL (100 and 200 mg/kg) treated groups, respectively. The average inhibitory rate of TriL on PCNA was 40.14% (100 mg/kg) and 64.28% (200 mg/kg) compared with the control group (*** *p* < 0.001) (Figure 4F).

### 2.3. TriL Inhibited PDGF-BB-Stimulated A7r5 VSMC Migration

**Figure 5:**
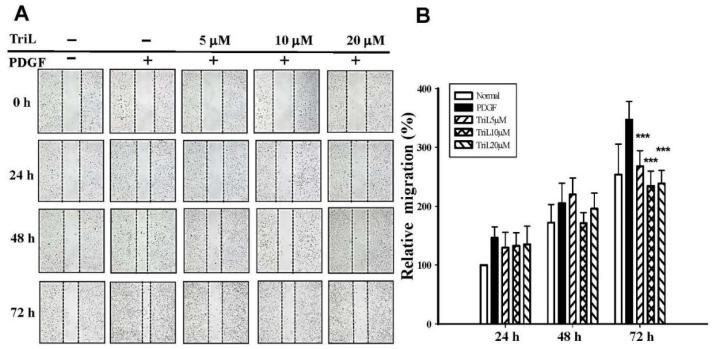
Effect of TriL on PDGF-BB-stimulated VSMC migration by wound healing assay. (**A**) A typical trace of TriL (5 μM, 10 μM, 20 μM) inhibition in response to PDGF-BB stimulated A7r5 VSMC migration. (**B**) Statistical differences in 24, 48, and 72 h at different TriL concentrations, respectively. The normal group was treated with vehicles. *** *p* < 0.001 compared with the PDGF-BB control group (n = 6).

The effects of TriL on 30 ng/mL PDGF-BB-stimulated A7r5 VSMC migration were evaluated using a wound healing assay. A7r5 cell monolayers were scratched, treated without (normal) or with PDGF-BB (30 ng/mL), and incubated without (normal) or with TriL (5 μM, 10 μM, 20 μM) for 24 h to 72 h. Figure 5A showed that 5–20 μM TriL inhibited PDGF-BB-stimulated A7r5 migration. Percentage of inhibition after 72 h of TriL treatment (5, 10, and 20 μM) were 22.89, 32.46%, and 32.25%, *** *p* < 0.001, respectively, and the inhibitory percentages of TriL (20 μM) were 13.39%, 21.29% and 32.25% at 24, 48, and 72 h, respectively (Figure 5B). 

**Figure 6:**
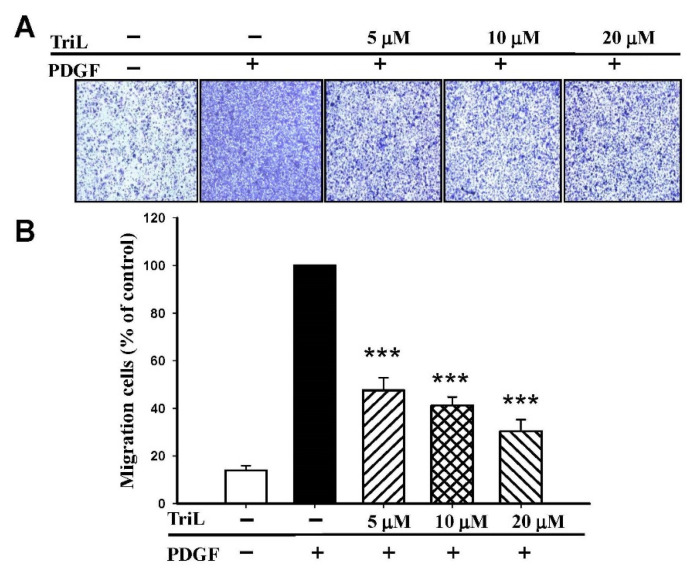
Effect of TriL on PDGF-BB-stimulated VSMC migration by transwell assay. (**A**) Inhibition of TriL (5, 10, and 20 µM) in response to PDGF-BB-stimulated VSMC migration. (**B**) Statistical difference of TriL (5, 10, and 20 µM) in response to PDGF-BB stimulated VSMC migration at different TriL concentrations. The normal group was treated with vehicles. *** *p* < 0.001 compared with the PDGF-BB control treated group (n = 6).

The effects of TriL on 30 ng/mL PDGF-BB-stimulated A7r5 migration were further evaluated by transwell assay. The ability of A7r5 cells to move across the membrane was significantly reduced by TriL treatment (Figure 6A). The migrated cell numbers induced by PDGF-BB across the membrane were significantly inhibited by TriL treatment. The inhibitory percentages were 52.34%, 58.78%, and 69.62% by TriL (5, 10, and 20 μM) compared with a PDGF positive control group *** *p* < 0.001 (Figure 6B).

### 2.4. TriL Inhibited PDGF-BB-Stimulated A7r5 VSMC Migration via Modulation of Ras/MEK/ERK Signaling Pathway and MMP-2 Protein Expression Level

**Figure 7:**
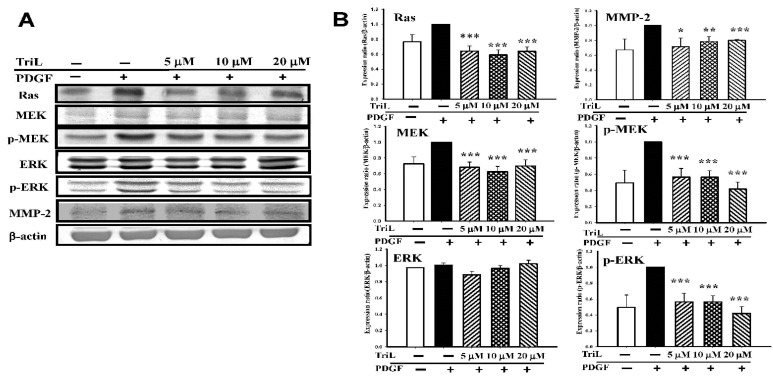
Effects of TriL on Ras/MEK/ERK pathway and matrix metalloproteinase-2 (MMP-2) protein levels. A series of experiments were performed to measure the effects of TriL on levels of the candidate signaling proteins. (**A**) Ras, MEK, p-MEK, ERK, p-ERK, and MMP-2 in PDGF-BB-stimulated A7r5 cells. (**B**) TriL at a concentration of 5, 10, 20 µM significantly reduced protein levels of Ras (*** *p* < 0.001); MEK (*** *p* < 0.001); p-MEK (*** *p* < 0.001) and p-ERK (*** *p* < 0.001). MMP-2 protein levels were also reduced by TriL (5, 10, 20 µM) * *p* < 0.05, ** *p* < 0.01, *** *p* < 0.001, respectively (n = 3~5).

A series of Western blotting was performed to measure the effects of TriL on levels of VMSC migration-related candidate signaling proteins, including Ras, MEK, p-MEK, ERK, p-ERK, and MMP-2. Various concentrations of TriL significantly reduced protein abundance of Ras, MEK, p-MEK, and p-ERK (Figure 7A). Percentages reduction in TriL (5, 10, and 20 μM) on protein levels were as follows: Ras were 31.83%, 37.33%, and 30.17%, respectively (*** *p* < 0.001); MEK were 20.67%, 15.67%, and 8.17%, respectively (*** *p* < 0.001); p-MEK were 43.60%, 43.80%, and 58.40%, respectively (*** *p* < 0.001); p-ERK were 44.80%, 52.20%, and 64.20%, respectively (*** *p* < 0.001). TriL (5, 10, and 20 μM) reduced the expressions of MMP-2, and the reduction rate on MMP-2 were 21.00%, 18.40%, and 12.00%, respectively (* *p* < 0.05, *** *p* < 0.01, *** *p* < 0.001) (Figure 7B).

## 3. Discussion

In this study, our data indicate that TriL significantly alleviated I/R-induced brain injury and carotid artery ligation-induced neointimal hyperplasia in vivo and reduced PDGF-BB-stimulated VMSC migration by modulating the Ras/MEK/ERK signaling pathway and MMP-2 in vitro. The distinct findings reveal that TriL protects against I/R injury and suppresses neointimal hyperplasia. Furthermore, it implies TriL may have therapeutic potential for neuroprotection and treating cerebrovascular diseases. Previous studies have reported that TriL has a myocardial protective effect, which is related to its antioxidation that decreases endogenous superoxide dismutase (SOD) [21,29] and the anti-inflammatory effects on lipopolysaccharide (LPS)-stimulated RAW 264.7 cells via the inhibition of NF-kB and MAPKs pathway [28]. A profound neuro-inflammation is induced in cerebral ischemia [30], triggers an inflammatory cascade in the injured brain, and contributes to neuron apoptosis and the development of brain injury [31]. Inhibition of neuro-inflammation in the ischemic brain is critical for neuroprotection and may provide clues for drug design in treating the cerebral injury. In this study, TriL dose-dependently ameliorated I/R-induced cerebral injury, including reduced infarct size and functional deficits, and mitigated neuronal apoptosis, revealing the neuroprotective effect of TriL on cerebral ischemia partially via the anti-oxidative and anti-inflammatory effects.

Atherosclerosis is one of the critical causes of cerebrovascular diseases, such as cerebral ischemia and stroke in the carotid and vertebrobasilar circulatory system [32]. In response to atherosclerosis or injury, blood vessels usually display intimal thickening, which leads to vessel occlusion and cerebral infarction [33]. Intimal hyperplasia involves inflammatory cells, their mediators, VSMC proliferation, and migration [34,35]. In addition, the balance of VMSC migration and proliferation over apoptosis can impact the final size of intimal thickening [8]. In the present study, TriL treatment showed a dose-dependent inhibitory effect on carotid artery ligation-induced intimal hyperplasia and reduced PCNA expression in the neointima, which implies TriL may have therapeutic potential for cerebrovascular diseases.

Abnormal VSMC proliferation is associated with the development of cerebrovascular diseases [36]. PDGF-BB, the most potent cell proliferation and migration stimulator, initiates cellular responses by activating intracellular signal transduction pathways such as mitogen-activated protein kinase/extracellular signal-regulated kinase (MAPK/ERK), leading to VSMC proliferation and migration [37,38]. The small G protein/mitogen extracellular signaling regulated kinase/extracellular signal-regulated kinase (Ras/MEK/ERK) signaling pathway in cardiovascular disease and I/R injury [38]. ERK is a crucial member of the MAPK family and participates in intracellular signaling transduction. Various stimuli induce ERK phosphorylation (p-ERK) in vivo and in vitro, and p-ERK moves to the nucleus, activating many transcription factors and regulating gene expression [39]. Abnormal activation of the ERK signaling pathway is closely related to developing I/R injury in various models [40,41], and ERK has been targeted to prevent and treat I/R injury [42]. Additionally, modulating VSMC proliferation has important therapeutic implications, and drug or gene therapy inhibition of the activated MAPK/ERK pathway can reduce neointimal hyperplasia [43]. The present study revealed that TriL treatment (5, 10, 20 µM) inhibited protein expressions of Ras, MEK, p-MEK, p-ERK, and MMP-2 in PDGF-BB-stimulated VSMCs and showed a significant effect even in a lower dose (5 µM) indicating TriL plays an essential role in the inhibiting of neointimal hyperplasia. Moreover, previous reports indicated that MMPs regulate the activity of VSMC in vitro and in vivo [8,17,18], induce VSMC migration [44], and are up-regulated in human atherosclerotic plaques [45]. Mainly, MMP-2 plays an essential role in VSMC migration and neointima formation, and PDGF is indicated to activate VSMC migration by inducing MMP-2 expression [46]. This study indicated that TriL significantly reduced the protein level of MMP-2 in PDGF-BB-stimulated VSMCs. The above data imply the potential role of TriL in preventing cerebrovascular diseases.

The functional measurement, such as infarction area, TUNEL-positive cells, and intimal hyperplasia induced by carotid-ligation, VSMC migration by wound healing assay, or transwell assay, is related to complex interactions between different signaling pathways. TriL treatment dose-dependently reduced the cerebral infarction area; neurological deficits and carotid-ligation induced intimal hyperplasia in vivo and inhibited VSMC migration in vitro. TriL significantly reduced the severity of intimal hyperplasia in a carotid-ligation model and reduced the PCNA-positive cells. The PCNA, as an index of cell proliferation, has been identified by immunofluorescence [47]. During atherosclerosis and intimal hyperplasia formation, various cytokines and growth factors stimulate VSMC to express MMPs [31,36]. In this study, TriL reduced the severity of intimal hyperplasia induced by carotid-ligation and decreased PCNA expression and PDGF-BB-stimulated VSMC migration. These inhibitory effects might be due to decreasing the activities of MMP-2 and modulating the Ras/MEK/ERK signaling pathway. The above data reveal that TriL is protective in models of I/R-induced brain injury, carotid artery ligation-induced intimal hyperplasia, and VSMC migration both in vivo and in vitro. TriL could be potentially efficacious in preventing cerebral ischemia and cerebrovascular diseases.

## 4. Materials and Methods

### 4.1. Chemicals and Reagents

The chemicals were purchased from the following companies. Trilionlein, PDGF-BB, and MTT (3-[4,5-dimethylthiazol-2-yl]-2,5-diphenyl tetrazolium bromide), and 2,3,5-triphenyltetrazolium (TTC) were from Sigma-Aldrich (St. Louis, MO, USA). The BCA assay kit (Pierce Biotechnology Inc., Rockford, IL, USA). Anti-Ras (ab16795), anti-MEK (ab178876), anti- phosphor-MEK1/2 (ab194754), anti-ERK (ab184699), anti-phospho-ERK1/2 (ab 65142), and anti-MMP-2 (ab37150) were from Abcam (Cambridge, UK). Anti-β-actin was from Santa Cruz Biotechnology (Santa Cruz, CA, USA). DMEM, penicillin/streptomycin, FBS, and glutamine were from ThermoFisher (Thermo Fisher Scientific Inc., Waltham, PA, USA). Zoletil^®^ was purchased from Virbac Laboratories (Carros, France). In Situ Cell Death Detection Kit, Fluorescein, following the manufacturer’s instructions (Merck KGaA, Darmstadt, Germany). DAPI (4′,6-diamidino-2-phenylindole, Thermo Fisher Scientific Inc., Waltham, PA, USA).

### 4.2. Animals

Male Sprague–Dawley (SD) rats weighing 220–270 g and male ICR mice weighing 22–25 g were purchased from BioLASCO Co. Ltd. (Taipei, Taiwan). All animals were fed with regular chow and housed in standard cages at a constant temperature of 22 ± 1 °C with a 12 h inverted light–dark cycle for one week before the experiments. Surgery was performed under zoletil^®^ anesthesia. Three to six animals were used to obtain consistent data in each group. This study reviewed and approved the animal experiment protocol by the Institutional Animal Care and Use Committee (IACUC), China Medical University (CMU-IACUC101-148-N).

### 4.3. Transient Focal Cerebral Ischemia/Reperfusion Model

Cerebral ischemia/reperfusion was induced by 3-vessels-occlusion; both common carotid arteries (CCA) and the middle cerebral artery (MCA), as previously described [48]. Take an appropriate amount of TriL and mix it with saline to the required concentration for animal experiment. Rats were randomly assigned to 4 groups (n = 5). Group 1 rats were treated with saline, and groups 2 to 4 were treated with TriL (50 mg/kg, 100 mg/kg, and 200 mg/kg, respectively). TriL (50 mg/kg, 100 mg/kg, and 200 mg/kg) was orally administered 1 h prior to transient focal cerebral ischemia-reperfusion (I/R). Animals were anesthetized with zoletil^®^ (25 mg/kg, i.p.). We placed the rat supine with a midline incision in the neck to expose both common carotid arteries and tied off both common carotid arteries with a plastic line (0.1 mm in diameter). Distilled water and different concentrations of TriL (50, 100, 200 mg/kg) were orally administered, respectively, 60 min prior to 3-vessels occlusion in rats. All rats were alive after 90 min occlusion followed by reperfusion for 24 h, and neurobehavioral evaluation and infarct assessment was performed. After transcardiac perfusion of 0.9% NaCl, the rat brain was removed, and each brain was coronally sectioned into 2 mm slices. The brain slices were stained with 2% TTC solution at room temperature for 15 min. The slices were fixed with a formalin solution (10%). The cerebral infarction areas of the frontal lobe were measured using the Image-Pro Plus 6.0 (Media Cybernetics, Rockville, MD, USA). The relative ratio of infarction area to total brain area was calculated, and the data were expressed as a percentage (%).

### 4.4. Immunofluorescence Staining of Apoptotic Cells

Rats were assigned randomly into four treatment groups (n = 5). Group 1 rats were treated with saline, and groups 2, 3, and 4 were treated with TriL (50 mg/kg, 100 mg/kg, and 200 mg/kg, respectively). Focal ischemia was induced by occlusion of both CCA and the right MCA, as previously described [48]. Twenty-four hours after focal ischemia and reperfusion, the immunofluorescence staining was performed. Rats were anesthetized with zoletil^®^ (50 mg/kg, i.p.). Intracardiac perfusion with 200 mL of 0.9% saline and 4% paraformaldehyde in 0.1 M PBS, then animals were decapitated [49].

The immunofluorescence staining of the brain slices was performed using the In Situ Cell Death Detection Kit, Fluorescein (Merck), following the manufacturer’s instructions manual. Then, the slide was counterstained by DAPI. Images were captured with a fluorescence microscope. The percentage of TUNEL/DAPI-positive apoptotic cells within the cortex were estimated based on the average number of cells in a defined area.

### 4.5. Carotid Ligation Model and Hematoxylin-Eosin Staining and PCNA Antibody Staining of the Carotid Artery

Male ICR mice (22 to 25 g) were randomly assigned to three treatment groups, with five mice for each group. On the day of ligation, all mice were anesthetized with 25 mg/kg zoletil^®^ (i.p.). Carotid ligation was performed according to the previous reports [50]. A midline neck incision exposed the left common carotid artery, and the carotid artery was ligated entirely just proximal to the carotid bifurcation, and the right carotid artery served as a non-injured control artery. All animals were allowed to recover after the incision was closed. Group 1 mice were treated with saline, and groups 2 and 3 were treated with 100 mg/kg and 200 mg/kg TriL, respectively. TriL and saline treatments were given by gastric gavage on the second day after carotid ligation, once daily for 28 days. These animals were euthanized on Day 29, and both common carotid arteries were harvested, and the arterial sections (5 µm) dehydrated in xylene and ethanol and embedded in paraffin for histomorphometric analysis using the H-E (hematoxylin-eosin) solution as previously described [49]. The images were digitized and analyzed with Image-Pro 6.0 software. The lumen areas, IEL (internal elastic lamina) and EEL (external elastic lamina), were determined using computerized planimetry. Subtracting the luminal from the IEL area to obtain the intima area and subtracting the IEL area from the EEL area to determine the media area [50]. In addition, the intima to media area (I/M ratio) was calculated and analyzed. Finally, arterial sections of animals from the different treatment groups were stained using an immunofluorescence method to detect PCNA (proliferating cell nuclear antigen) [47].

### 4.6. Vascular Smooth Cell Line and In Vitro Wound Healing Assay

A7r5, a VSMC cell line, was purchased from Bioresource Collection and Research Center, Hsinchu, Taiwan. A7r5 cells were plated onto 6-well plates in 10% FBS, 2 mM L-glutamine, 100 unit/mL penicillin, and 100 mg/mL streptomycin-supplemented DMEM. A7r5 cells were grown at 37 °C under a humidified 5% CO_2_ and 95% air in one atmosphere. For A7r5 cells, trilinolein was dissolved in 0.8% (*v*/*v*) Tween 80 in PBS and sterilized by filtration, then diluted to the appropriate concentrations with a cell culture medium. The effects of TriL on VSMC migration were evaluated using the wound-healing assay. A7r5 cells were plated in 6-well plates with 2 × 10^5^ cells in each well, and a single scratch wound was performed using a sterile micropipette tip as previously described. Cells were then incubated with or without 30 ng/mL PDGF-BB and TriL (5, 10, 20 µM) in a DMEM medium (containing 0.5% fetal bovine serum). A phase-contrast microscope was used to determine the extent of wound closure at 24, 48, and 72 h following wounding. According to the previous report [50], the migration distance of drug-treated cells expressed cell migration (mm) divided by the migration distance of untreated cells (mm).

### 4.7. Transwell Migration Assay

The effects of TriL on VSMC migration were further investigated using a transwell migration chamber containing a collagen-coated polycarbonate filter as previously described [50]. 2 × 10^5^ A7r5 cells were seeded on a transwell apparatus, a 6.5-mm polyethylene terephthalate membrane with 8-µm pores, purchased from Millicell (Merck KGaA, Darmstadt, Germany), and treated with TriL (5, 10, 20 µM) in the presence of 30 ng/mL PDGF-BB for 48 h. A7r5 cells were trypsinized and resuspended in a 0.5% FBS medium. 10% FBS/DMEM was added to the bottom chamber of each well as the chemoattractant. Cells were allowed to migrate for 8 h through the membrane to the underside of the apparatus; then, cells were fixed with methanol for 10 min and stained with Giemsa solution for 30 min. Cell migration to the lower outside of the insert membrane was counted manually using a microscope and the NIS-Elements software (Nikon Inc., Melville, NY, USA).

### 4.8. Protein Preparation and Western Blot Analysis

The role of the Ras/MEK/ERK signaling pathways and the matrix metalloproteinase-2 were used to evaluate the effect of TriL on VSMC migration. 5 × 10^6^ A7r5 cells were seeded in a 10-cm culture dish and then treated with 30 ng PDGF-BB and TriL (5, 10, 20 µM) for 48 h. A7r5 cells were harvested and washed with cold 1x PBS. Total protein was collected, and the concentration was measured using the BCA assay kit. The cell lysate was run on a 10% SDS-polyacrylamide gel electrophoresis (SDS-PAGE) with an equal volume and electrotransferred to a polyvinylidene fluoride (PVDF) membrane (Thermo Fisher Scientific Inc., Waltham, PA, USA) by using iBot^TM^ Gel Transfer System (Thermo Fisher Scientific Inc., Waltham, PA, USA). The blot was soaked in blocking buffer (5% non-fat dry milk/0.05% Tween 20 in 20 mM TBS at pH 7.6) at room temperature for 1 h and then incubated with anti-Ras (1:1000), anti- MEK (1:20,000), anti-phosphor-MEK1/2 (1:1000), anti-ERK (1:10,000), anti-phospho-ERK1/2 (1:1000), anti-MMP-2 (1:2000), and β-actin antibodies in blocking buffer, respectively, at 4 °C overnight as previously described [50]. Membranes were washed with Tris-buffered saline/Tween 20 three times for 10 min and then incubated with secondary horseradish peroxidase (HRP)-conjugated antibody. The blots were developed using chemiluminescence (ECL) detection kit (Thermo Fisher Scientific Inc., Waltham, PA, USA) as previously described [50]. β-Actin was used as an internal loading control.

### 4.9. Statistical Analysis

Data are expressed as mean ± SE. Differences among groups were analyzed using ANOVA (one-way analysis of variance) followed by Scheffe’s test for individual comparisons. When a *p*-value < 0.05 was considered statistically significant.

## 5. Conclusions

**Figure 8:**
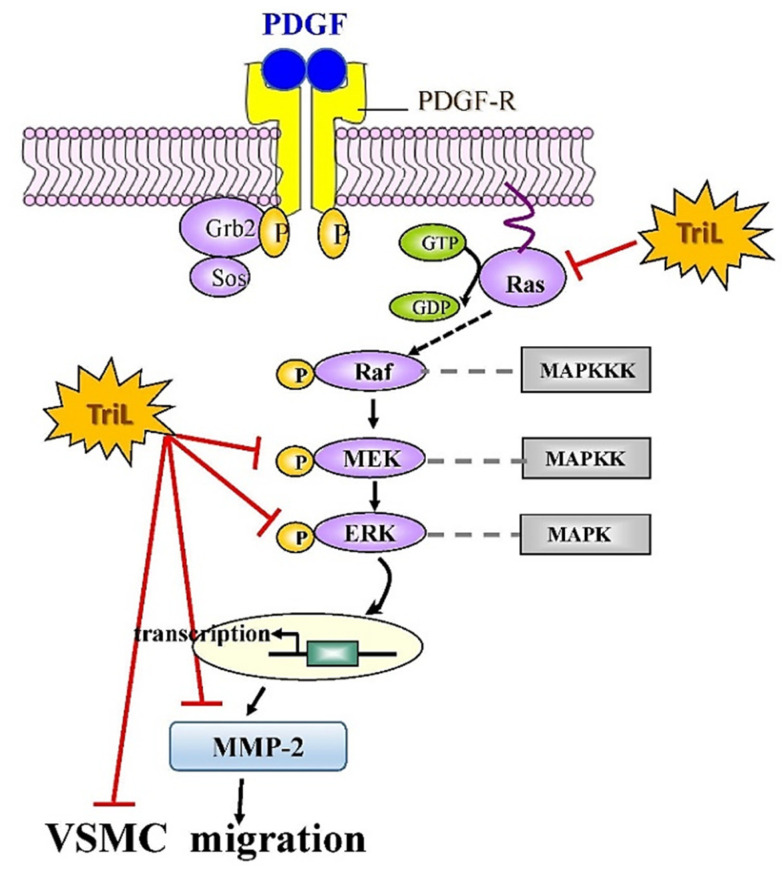
Proposed mechanisms of TriL inhibited PDGF-BB-stimulated A7r5 VSMC migration via modulation of Ras/MEK/ERK and MMP-2 protein expression. Ras, small G protein; Raf, serine/threonine kinase; MEK, mitogen extracellular signaling regulated kinase; ERK, extracellular-signal-regulated kinase; MMP-2, matrix metalloproteinase-2; MAPKKK, mitogen-activated protein kinase kinase kinase; MAPKK, mitogen-activated protein kinase kinase; MAPK, mitogen-activated protein kinase; –l, Inhibitory modulation.

Our findings indicate that TriL is protective in models of I/R-induced brain injury, carotid artery ligation-induced intimal hyperplasia in vivo and PDGF-BB-stimulated VSMC migration in vitro. These findings suggest that TriL might be a therapeutic candidate for preventing cerebrovascular disease in high-risk individuals and could help treat patients with cerebrovascular disease. In addition, a proposed mechanism of TriL inhibited PDGF-BB-stimulated A7r5 VSMC migration via modulation of the Ras/MEK/ERK signaling and MMP-2 and protein expression is shown in Figure 8.

## Data Availability

Data generated or analyzed in this published article are all included.
